# Electrorheological Properties of Polydimethylsiloxane/TiO_2_-Based Composite Elastomers

**DOI:** 10.3390/polym12092137

**Published:** 2020-09-18

**Authors:** Alexander V. Agafonov, Anton S. Kraev, Alexander E. Baranchikov, Vladimir K. Ivanov

**Affiliations:** 1 Krestov Institute of Solution Chemistry, Russian Academy of Sciences, 153045 Ivanovo, Russia; a.s.kraev@mail.ru; 2 Kurnakov Institute of General and Inorganic Chemistry, Russian Academy of Sciences, 119991 Moscow, Russia; a.baranchikov@yandex.ru (A.E.B.); van@igic.ras.ru (V.K.I.); 3 Higher School of Economics, National Research University, 101000 Moscow, Russia

**Keywords:** crosslinking, TiO_2_, nanomaterials, stimuli-responsive materials, smart materials

## Abstract

Electrorheological elastomers based on polydimethylsiloxane filled with hydrated titanium dioxide with a particle size of 100–200 nm were obtained by polymerization of the elastomeric matrix, either in the presence, or in the absence, of an external electric field. The viscoelastic and dielectric properties of the obtained elastomers were compared. Analysis of the storage modulus and loss modulus of the filled elastomers made it possible to reveal the influence of the electric field on the Payne effect in electrorheological elastomers. The elastomer vulcanized in the electric field showed high values of electrorheological sensitivity, 250% for storage modulus and 1100% for loss modulus. It was shown, for the first time, that vulcanization of filled elastomers in the electric field leads to a significant decrease in the degree of crosslinking in the elastomer. This effect should be taken into account in the design of electroactive elastomeric materials.

## 1. Introduction

The electrorheological effect is a reversible and rapid change in the physicomechanical properties of a composite dielectric material when an external electric field is applied [1,2]. Materials exhibiting the electrorheological effect, and their analogues–magnetorheological materials, are typical representatives of smart materials that are extremely promising for application in various industrial devices, including vibration absorbers, dampers, etc. [3,4].

Depending on the type of dielectric dispersion medium, electrorheological fluids (liquid dispersion medium) and electrorheological gels and elastomers (solid dispersion medium) are distinguished [5,6]. In electrorheological fluids, the electrorheological effect is caused by the movement of particles of the polarizable disperse phase in an electric field, with the formation of ordered structures and the transition of the dispersed system from a viscous flowing state to a viscoplastic state [7,8]. The main disadvantage of electrorheological fluids is the lack of long-term stability and gradual sedimentation of particles of the disperse phase. Despite the fact that a strong electrorheological effect was observed for suspensions containing particles of a disperse phase with a high density (up to 7 g/cm^3^) [9,10,11,12], providing sedimentation stability of electrorheological fluids usually requires the use of additional stabilizers or constant mixing of the suspension.

In electrorheological elastomers, the mobility of the particles of the disperse phase (filler particles) is hindered by the polymer matrix, so there is no problem of sedimentation stability in such systems. Unfortunately, almost complete immobility of the filler particles does not allow for restructuring of the electrorheological elastomer when an electric field is applied, and so in these materials the electrorheological effect arises due to dipole-dipole interactions between isolated particles of the disperse phase [13].

A number of studies have been devoted to the creation of electrorheological elastomers, covering the use of various fillers and polymer compositions, aimed at the development of electrorheological elastomers with improved dynamic characteristics and mechanical properties, and the development of model representations to describe the electrorheological and viscoelastic mechanical behavior of elastomers, as well as the search for possible areas of practical application of electrorheological elastomers [14,15,16,17,18,19,20]. Despite some advances in the field of electrorheological elastomers production, the design of electrorheological elastomers with large-scale changes in viscoelastic properties in electric fields is currently difficult, due to the lack of a generally accepted behavior theory for such disperse systems. As a first approximation, the polarization theory is applied, which is generally used for the interpretation of the electrorheological effect in electrorheological fluids. Based on this theory, the key parameters determining the electrorheological effect are the electric field strength, the distance between the filler particles, the dielectric relaxation time as well as the dielectric constant, the dielectric loss tangent and the electrical conductivity of the materials of the filler particles, and the dispersion medium [3,21,22,23,24,25,26,27]. The surface chemical composition of particles in the disperse phase [28,29,30], the presence of the regions with a reduced viscosity in the dispersion medium [31], as well as the nature of the distribution of filler particles in the matrix, can also make a significant contribution to the formation of the electrorheological effect.

The most well-known and simple method to organize the structure of electrorheological elastomers is the polymerization of dielectric suspensions in a constant electric field [13]. During the polymerization, ordered structures are preserved, resulting from the arrangement of polarized particles of the disperse phase in a liquid. As a rule, the electrorheological effect for structured elastomers is greater than for materials with a stochastic distribution of filler particles, which is associated with the smaller distance between the particles of the disperse phase. Anisotropically-filled elastomers are also characterized by a higher dielectric constant and Maxwell–Wagner dispersion [32,33,34].

At the same time, the factors determining the magnitude of the electrorheological response of filled elastomers remain largely unexplored. In our opinion, the strength of contact between the particles of the disperse phase and the dispersion medium is of great importance, as well as the presence of low molecular weight compounds on the surface of the particles, which act as activators of the electrorheological effect. In particular, during the curing of the elastomer in the electric field, regions with different degrees of polymerization of the elastomer may appear, which could unpredictably affect the physicomechanical properties of the material.

The aim of this work is to analyze the dielectric and electrorheological characteristics of polydimethylsiloxane elastomers containing amorphous hydrated titanium dioxide vulcanized in, and in the absence of, an electric field.

## 2. Experimental Part

Titanium dioxide was synthesized by the hydrolysis of titanium tetraisopropoxide (#87560, Sigma-Aldrich, Darmstadt, Germany) in non-absolute ethanol. A 250-mL flask containing 50 mL of 95.6% ethanol was placed in a dry box and, with vigorous stirring, 12.7 g of Ti (O*i*Pr)_4_ was added dropwise to ethanol. The resulting white precipitate was kept in the mother liquor, under continuous stirring at room temperature, for 5 h, after which it was separated by centrifugation and dried to a constant mass in an oven at 60 °C.

To obtain a composite material containing titanium dioxide dispersed in a polydimethylsiloxane matrix, we used liquid siloxane rubber (polymerization degree, *n* = 100–5000) with terminal silanol groups (Vixint PK-68A compound, NPP Khimprom LLC, Yekaterinburg, Russia). Titanium dioxide powder was mixed with a siloxane liquid to obtain a composite containing 30 wt.% (~17 vol.%) TiO_2_, and the resulting suspension was mixed for 30 min, followed by the addition of 3 wt.% of the polymerization catalyst (a solution of aminopropyltriethoxysilane in ethyl silicate with a mass ratio of 1:4) [35] and vigorous stirring of the resulting mixture at 500 rpm for 5 min, after which the mixture was evacuated at 10^–2^ atm to remove air bubbles. The obtained suspension was placed in a cylindrical polymethylmethacrylate container with a diameter of 20 mm and depth of 2.5 mm, and was left to cure at room temperature. A mold with flat electrodes installed in its base was used for curing the suspension in the electric field. Within an hour of filling the mold, an alternating (10 Hz) voltage of 5 kV was applied to the electrodes. Complete curing of the elastomer was achieved within 5 h. Upon the vulcanization, the elastomers adhered firmly to the electrodes, eliminating possible electrode slip relative to the elastomer sample surface. Hereafter, a sample vulcanized in the absence of an electric field is designated ERE-0, and a sample vulcanized in an electric field at a voltage of 5 kV is designated ERE-5.

X-ray diffraction analysis (XRD) was carried out using a Bruker D8 Advance diffractometer (Karlsruhe, Germany) (CuKα radiation) in the angle range 5–80° 2θ, with a step of 0.02° 2θ and an accumulation duration of 0.2 s/step.

Scanning electron microscopy (SEM) was performed using a Tescan Vega 3 SBH microscope (Kohoutovice, Czech Republic) at an accelerating voltage of 5 kV.

IR spectra were recorded using a Bruker VERTEX 80v IR Fourier spectrometer (Karlsruhe, Germany). The FTIR reflection spectra were recorded in the region of 400 to 4000 cm^−1^, with a resolution of 2 cm^–1^, at room temperature.

Low-temperature nitrogen adsorption analysis was performed using a Quantachrome NOVAtouch NT LX-specific surface and porosity analyzer (Boynton Beach, FL, USA).

The dependences of the dielectric constant and dielectric loss tangent on the frequency of the electric field of composite elastomers were measured in a capacitor-type cell with spring-loaded disk plane-parallel electrodes made of polished stainless steel, using a Solartron SI 1260 Impedance/Gain-Phase analyzer (Farnborough, United Kingdom) in the frequency range 25–10^6^ Hz at 1 V voltage. The effect of the electric field strength on the dielectric characteristics of filled elastomers was analyzed in the same cell, using an Electronpribor MEP-4CA Schering Bridge (Moscow, Russia) with a reference capacitor at a frequency of 50 Hz in the voltage range 0.2–1.0 kV. All measurements were performed at room temperature.

To conduct electrorheological measurements, a rheometer operating in the controlled shear deformation mode was used with a stepper motor with a controlled rotation speed and a torque measuring system. A cell with two parallel plates of polished brass with a diameter of 20 mm and an adjustable gap was used for all measurements. A voltage up to 5.0 kV was generated between the upper movable plate and the lower plate connected with the strain gauge; after 60 s, the rheometer drive was turned on and the measured torque values were recorded automatically every 0.1 s at a shear rate of 0.1 rad/s until shearing angle reached 0.192 rad. After the measurement, the rheometer plate was returned to a starting position. The absence of the electrode slip relative to the sample surface was checked by the coincidence of marks applied on the side faces of the electrodes and the elastomer sample.

## 3. Results and Discussion

**Figure 1:**
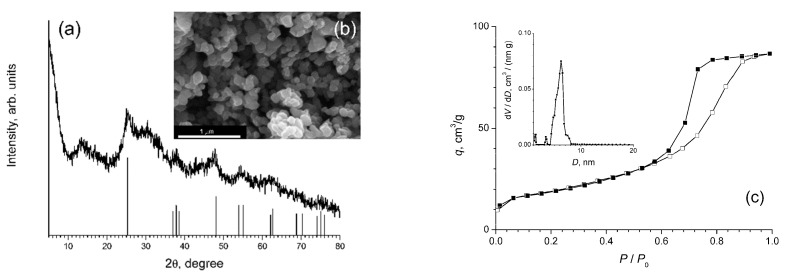
The analysis results of titanium dioxide powder obtained by hydrolysis of titanium isopropoxide in ethanol, using (**a**) XRD (Bragg positions correspond to anatase); (**b**) SEM; (**c**) low-temperature nitrogen adsorption. The inset shows the pore size distribution.

Figure 1 shows the results of the analysis (XRD, SEM, low temperature nitrogen adsorption) of titanium dioxide powder obtained by hydrolysis of titanium isopropoxide in ethanol. The titanium dioxide powder was almost completely X-ray amorphous; the diffraction pattern (Figure 1a) contained weak reflections (in particular, ~25.7° 2θ), which can be attributed to anatase [36]. The amorphous state is typical of titanium dioxide obtained by the sol-gel method without any further hydrothermal or thermal treatment [37,38]. At the same time, as previously noted in a number of studies, amorphous titanium dioxide can exhibit short-range order typical of crystalline polymorphic modifications of TiO_2_ (in particular, anatase), which determines the mechanism of its crystallization in aqueous media at elevated temperatures [39].

SEM data (Figure 1b) indicated a rather low degree of agglomeration of the obtained TiO_2_ powder, which consisted of relatively large (100–200 nm) particles of an almost isotropic shape. The specific surface area of the powder (65 m^2^/g) indicated that these particles were sufficiently dense, which was further confirmed by the fact that only small mesopores (4–8 nm) were present in the powder, while the larger mesopores, as well as micropores, were nearly absent. Capillary condensation hysteresis (Figure 1c), which belongs to type H2, according to the IUPAC classification (type E according to the de Boer classification) [40], indicated the presence of narrow, cylindrical pores.

The shear modulus [41] was used to describe the viscoelastic properties of elastomers, which is considered as the complex number $G{\;}^{*}=G^{\prime }+iG^{\prime \prime }$, where $G^{\prime }$ (storage modulus) is connected with the energy consumption for the deformation of the elastic structural elements in the elastomer, and $G^{\prime \prime }$ (loss modulus) characterises the energy dissipation into viscous losses.

**Figure 2:**
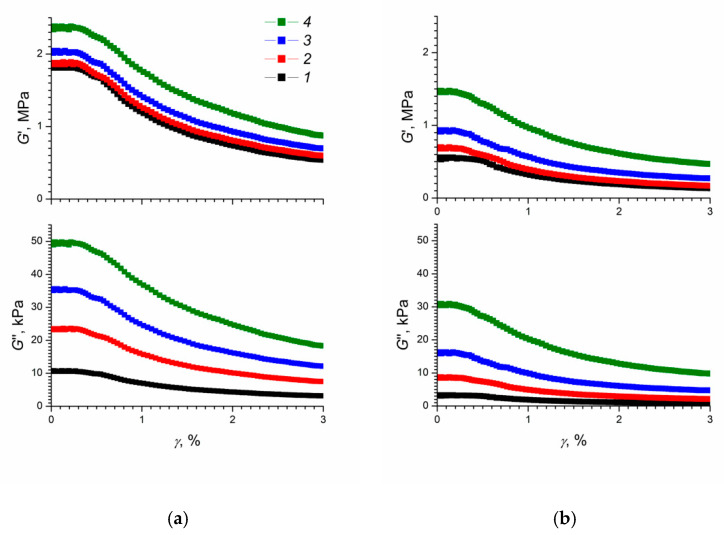
Storage modulus ($G^{\prime }$) and loss modulus ($G^{\prime \prime }$) as functions of relative deformation in electric fields of various strengths (1–0 kV/mm; 2–0.4 kV/mm; 3–1.2 kV/mm; 4–2 kV/mm) for elastomer samples modified with titanium dioxide and vulcanized (**a**) in the absence of an electric field and (**b**) in an electric field. The shear rate was 0.1 rad/s.

Figure 2 shows the storage and loss moduli of the composite elastomer obtained by vulcanization in the absence or in the presence of an electric field, as functions of the relative deformation in external electric fields of various strengths. For comparison purposes, the storage and loss moduli of bare elastomer samples obtained by vulcanization in the absence or in the presence of an electric field, as functions of the relative deformation are presented in Figure S2. Bare elastomer samples vulcanized in the absence or in the presence of an electric field (see Figure S2), show virtually no dependence on the storage and loss moduli on the relative deformation. The $G^{\prime }$ and $G^{\prime \prime }$ values for unfilled elastomers are lower than that of the filled ones, confirming higher elasticity and lower viscosity of the former under rotational strain.

The data presented in Figure 2 indicate that, in the absence of an electric field, the ERE-0 sample behaved as a highly elastic material; the storage modulus at a shear rate of 0.1 rad/s at low strain values was about 1.8 MPa. In turn, the storage modulus of the ERE-5 sample under the same conditions was about 0.5 MPa. Until a 0.5% degree of deformation was reached, the values of the storage modulus for both samples remained constant and decreased at large strains. This effect is similar to the well-known Payne effect (otherwise called the Fletcher-Gent effect) observed for filled elastomers [42,43] and which arises from the bonds breaking between filler particles [44]. The presence of the Payne effect indicates a high degree of interaction between the filler particles in the composite elastomer [45].

Previously, the Payne effect was detected in magnetorheological elastomers, and the magnitude of the effect increased when an external magnetic field was applied [46]. Our data confirm the results of a recent study [45] mentioning the Payne effect in polysiloxane-based electrorheological elastomers.

**Figure 3:**
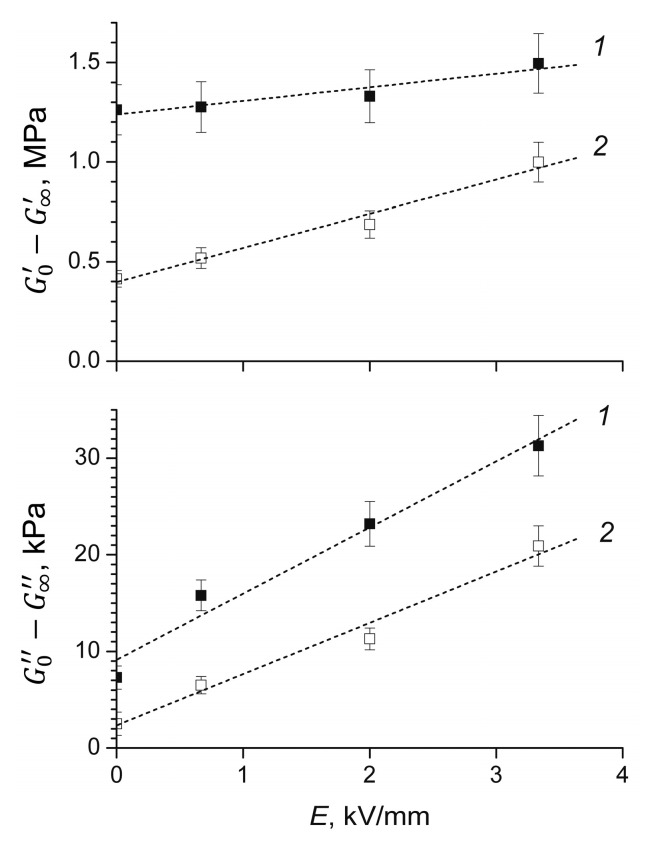
The effect of the external electric field strength on the value of the Payne effect for the storage modulus ($G_{0}^{\prime }-G_{\infty }^{\prime }$) and loss modulus ($G_{0}^{\prime \prime }-G_{\infty }^{\prime \prime }$) for the ERE-0 and ERE-5 samples.

It is noteworthy that, for ERE-0 and ERE-5 samples, the values of the storage and loss moduli remained constant at low strain values in the entire range of external electric field strengths (Figure 2). To determine the effect of the electric field strength on the value of the Payne effect in electrorheological elastomers based on polydimethylsiloxane, the values of the storage and loss moduli were estimated at zero and infinitely large deformations [42] and the corresponding differences were calculated, $G_{0}^{’}-G_{\infty }^{’}$ and $G_{0}^{’’}-G_{\infty }^{’’}$ (Figure 3).

One can note that the values of the Payne effect for the storage and loss moduli increased in an external electric field. This increase was apparently due to the fact that polarized filler particles, as a result of small spatial displacements (mainly rotation), contributed to an increase in the conformational mobility of macromolecules.

**Figure 4:**
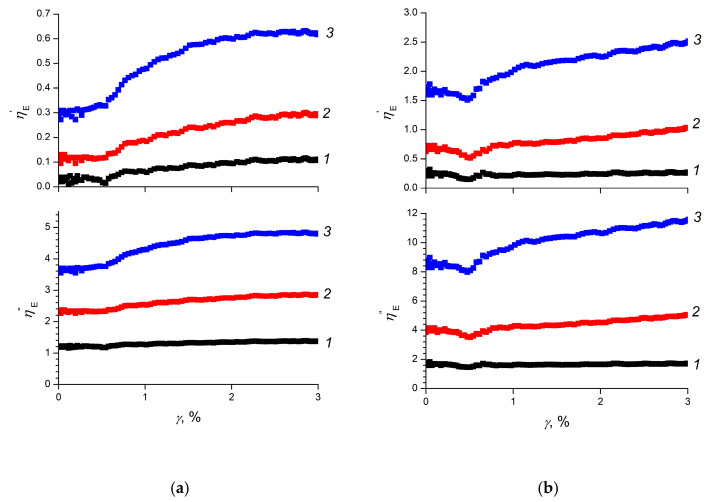
The electrorheological sensitivity of composite elastomers (**a**) ERE-0 and (**b**) ERE-5 with respect to storage ($\eta_{E}^{\prime }$) and loss ($\eta_{E}^{\prime \prime }$) moduli as function of relative deformation at various strengths of an external electric field (1–0.4 kV/mm, 2–1.2 kV/mm, 3–2.0 kV/mm).

The electrorheological sensitivity of the elastomers (the efficiency of converting the energy of an electric field into mechanical energy, $\eta_{E}$) can be estimated using the relative increments of the storage and loss moduli and in electric fields of different strengths (Figure 4) [25]:

(1) $$\eta_{E}^{’}=\frac{G_{E}^{’}-G_{0}^{’}}{G_{0}^{’}}\;\mathrm{and}\;\eta_{E}^{’’}=\frac{G_{E}^{’’}-G_{0}^{’’}}{G_{0}^{’’}}$$

where $G_{E}$ is a storage modulus or loss modulus of the elastomer in an electric field; $G_{0}$ is a storage or loss modulus of the elastomer in the absence of an electric field.

As follows from Figure 4a, the electrorheological sensitivity for the ERE-0 sample by storage modulus reached 63% in the 2.0 kV/mm electric field, which corresponds to the characteristics obtained for electrorheological composite elastomers filled with titanium dioxide [25,26]. Electrorheological sensitivity for the sample ERE-0 by the loss modulus reached 450% in the 3 kV/mm field.

The electrorheological sensitivity for the ERE-5 sample was significantly higher by both storage modulus and loss modulus (Figure 4b). In a 2.0 kV/mm electric field, the $\eta_{E}^{’}$ value reached 250%, and the $\eta_{E}^{’’}$ value exceeded 1100%. High values of electrorheological sensitivity for the ERE-5 sample indicate the high mobility of macromolecules in its structure and a significant range of changes in its elasto-plastic properties when an electric field was applied, comparable with the control range of magnetorheological elastomers [47]. It should be noted that the high electrorheological sensitivity of the ERE-5 sample, in comparison with the ERE-0 sample, is consistent with significantly higher values of the dielectric characteristics of the ERE-5 sample (see below)–the permittivity difference *ε*_0_–*ε*_∞_, higher tg*δ* values at the point of relaxation maximum and slower relaxation processes during polarization, which, according to general theories about the electrorheological sensitivity of electrorheological fluids, should contribute to a pronounced electrorheological effect.

A specific feature of the electrorheological elastomers ERE-0 and ERE-5 is their almost constant electrorheological sensitivity in the region of small deformations (up to *γ* ≈ 0.5%) at a fixed value of the external electric field. In the region of relative deformations *γ* > 0.5%, an increase in deformation led to an increase in electrorheological sensitivity by 1.5–2 times. These observations indicate that, with a relative deformation *γ* ≈ 0.5%, the polymer matrix was being rearranged, forming a relatively mobile viscoplastic structure. This effect allows for expanding the range of regulation of the physicomechanical properties of the composite elastomers using external electric fields.

The results obtained indicate that, in order to achieve high electrorheological sensitivity of composite elastomeric systems, an elastomer material must be preliminarily turned to a stressed state by a slight mechanical load. This stress can be caused by either external or internal, factors. In an electric field, filler particles immobilized in a polymer matrix are affected by polarizing forces that contribute to the rotation and displacement of particles relative to electric field lines. The movement of particles leads to the deformation of polymer molecules, primarily due to their conformational mobility, which in turn leads to an increase in the forced elasticity of the elastomer. The summation of these phenomena determines the features of the electrorheological effect in filled elastomers.

At the same time, these features cannot clarify the differences in the electrorheological behavior observed for the ERE-0 sample (vulcanized in the absence of an electric field and presumably characterized by a stochastic distribution of particles of the disperse phase) and ERE-5 sample (vulcanized in an electric field and presumably characterized by an anisotropic structure). In particular, the significantly higher electrorheological sensitivity with respect to loss modulus observed for the ERE-5 elastomer compared to the ERE-0 elastomer may have been due to the presence of a viscous flowing component in the structure of the former.

**Figure 5:**
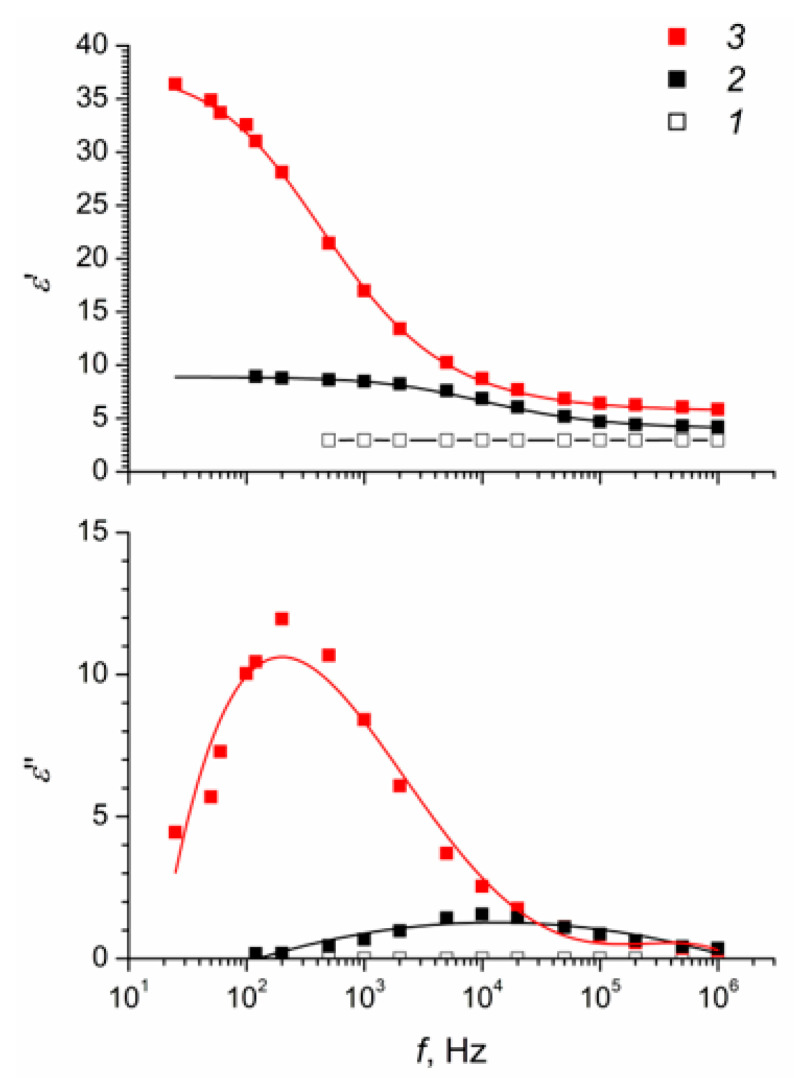
The dielectric constants (*ε*′, *ε*′′) as functions of frequency for (1) unmodified polydimethylsiloxane and TiO_2_-modified polydimethylsiloxane samples: (2) ERE-0; (3) ERE-5.

**Figure 6:**
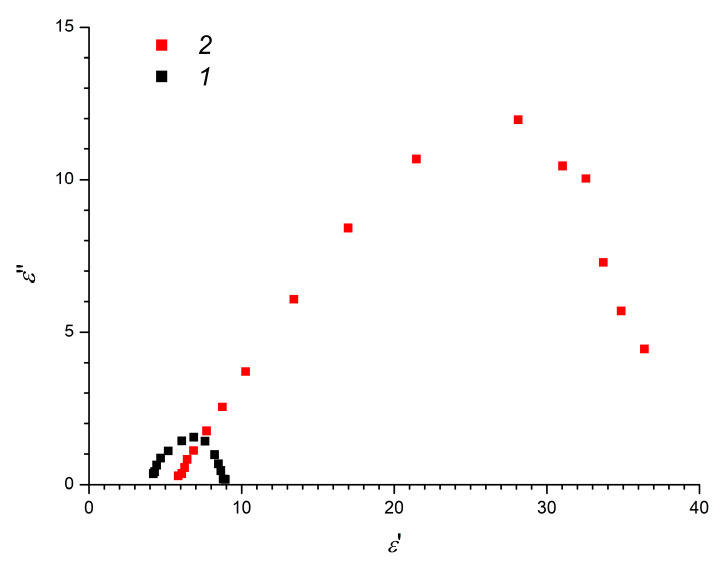
Cole–Cole diagrams for TiO_2_-modified elastomers: (1) ERE-0; (2) ERE-5.

To identify the nature of the differences in the electrorheological properties of the obtained composite elastomers, an analysis of their dielectric characteristics was performed (Figure 5 and Figure 6).

The dielectric characteristics of a filler-free elastomer (Figure 5) were frequency independent, while filled elastomers exhibited pronounced dependences of the dielectric constant and dielectric loss tangent on the frequency of the electric field. Comparison of the dielectric characteristics of filled elastomers (Figure 5) reveals that, for a sample vulcanized in the absence of an electric field, the nature of the relaxation processes was close to Debye and typical for systems with an isotropic structure. Conversely, for the asymmetry of the Cole–Cole diagrams (Figure 6), a larger range of changes in the dielectric constant and large values of the dielectric loss tangent for a composite elastomer vulcanized in an electric field indicates the presence of polarizable elements with different characteristic relaxation times.

Figure 5 and Table 1 show the results of fitting the dielectric spectra of the elastomer samples to the Havriliak–Negami Equation [48]:

(2) $$\varepsilon_{\omega }^{’}=\varepsilon_{\infty }+\frac{\varepsilon_{S}-\varepsilon_{\infty }}{{\left(1+{\left(i\;\omega \tau \right)}^{\alpha }\right)}^{\beta }}$$

where $\varepsilon_{\omega }^{’}$ is a dielectric permittivity at a circular frequency *ω*; $\varepsilon_{S}$–dielectric permittivity at zero frequency; $\varepsilon_{\infty }$–dielectric permittivity at infinite frequency; *τ*–dielectric relaxation time; *α*, *β*–parameters related to the distribution of relaxation times.

Table 1 shows that both ($\varepsilon_{\infty }-\varepsilon_{S}$) and *τ* values for the ERE-5 sample are notably higher than those for the ERE-0 sample, which is probably due to the different contribution of interphase polarization. Figure S3 shows the frequency dependences of the composite elastomer conductivity, which are typical to hopping charge transport mechanism in titanium dioxide prepared by sol-gel method [49]. ERE-5 sample demonstrates higher conductivity at frequencies below 55 kHz, while at higher frequencies, the sample has lower conductivity. The different efficiency of charge transfer process in these composites may be due to the different environment of titanium dioxide particles in the elastomer matrix.

The ordered arrangement of the particles of the disperse phase in the elastomer is not the only factor determining the dielectric characteristics of polymer composites, as the crosslinking density of macromolecules is also an extremely important parameter. For temperatures above the glass transition temperature of the polymer, the dielectric constant and dielectric loss tangent increase with decreasing crosslink density of the macromolecules. A decrease in the crosslinking density of polydimethylsiloxane macromolecules (for example, due to the introduction of plasticizers [50]) can be used for the advanced design of composites with improved electrorheological properties.

**Figure 7:**
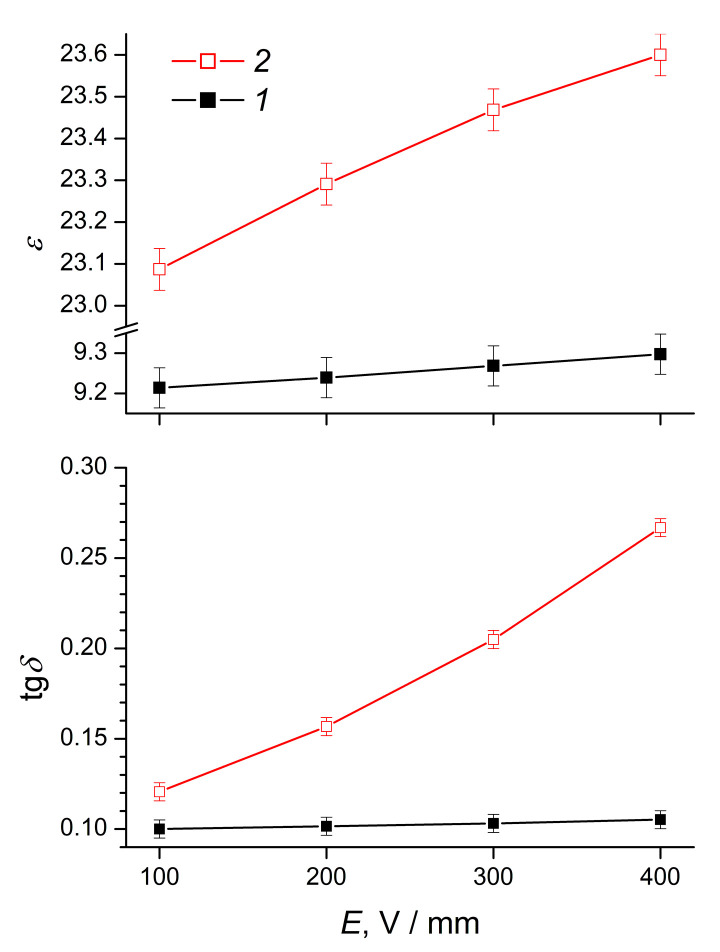
The dielectric constant (*ε*) and dielectric loss tangent (tg*δ*) of composite elastomers (1) ERE-0 and (2) ERE-5 as functions of the electric field strength.

From the analysis of the electric field strength effect on the dielectric characteristics of composite elastomers (Figure 7), it follows that in 0.1–0.4 kV/mm electric fields, the dielectric constant and the dielectric loss tangent were directly proportional to the field strength. With an increase in the field strength from 0.1 to 0.4 kV/mm, the change in *ε* value for the ERE-0 and ERE-5 samples was almost the same, amounting to 0.5–0.9, while the change in the dielectric loss tangent for the ERE-5 sample (~0.14) significantly exceeded the similar characteristic for the ERE-0 sample (~0.05). Since the conductivity can be derived from the dielectric characteristics of the material [51]:

(3) $$\sigma =\varepsilon_{0}\cdot \varepsilon \cdot \omega \cdot \mathrm{tg}\delta$$

where *ω* is a circular frequency, such a difference leads to a significantly larger increase in conductivity in the electric field of an ERE-5 sample, compared with an ERE-0 sample.

The higher conductivity of the composite elastomer ERE-5 can be caused by various factors. First, as a result of vulcanization of the composite elastomer in an external electric field, aggregates of disperse phase particles oriented in the direction of the electric field form in the volume of the polymer, along which charge transfer can occur [52]. Obviously, with an equal content of the disperse phase, the percolation threshold for composites containing oriented particle aggregates will be lower than for a composite with a stochastic distribution of filler particles. At the same time, high dielectric characteristics and, consequently, relatively high conductivity of elastomeric materials can be caused by the presence of mobile structural elements, for example, low molecular weight compounds or polymer molecules, with a lower average molecular weight compared to the main polymer component.

In order to identify the possible contribution of the latter factor to the electrorheological and dielectric properties of the obtained elastomers, we assessed a degree of polymer crosslinking in composites vulcanized in an electric field or without it. The crosslinking degree was evaluated gravimetrically by determining the degree of swelling [53,54].

For this, samples of elastomers filled with titanium dioxide were immersed in toluene and left to swell for one day, with stirring. After this, toluene was replaced by a different volume of toluene and the procedure was repeated for an additional day. After swelling, the samples were thoroughly washed with ethanol and dried under reduced pressure (10^–2^ atm) at 50 °C.

The following equations were used for calculations:

(4) $$\beta =\frac{m_{H}-m_{t}}{m_{t}}\cdot 100\%$$

(5) $$\omega_{c}=\frac{m_{0}-m_{t}}{m_{0}}\cdot 100\%$$

Here, *β* is the swelling degree; *ω*_c_ is the mass fraction of components not polymerized into a common network; $m_{0}$ is the initial mass of the composite elastomer; *m*_H_ is the mass of swelled composite elastomer; $m_{t}$ is the mass of elastomer after washing with ethanol and drying. The weight of titanium dioxide contained in the composite sample was subtracted from the values $m_{0}$, *m*_H_, $m_{t}$ before calculations. The Flory–Rehner equation was used to determine the degree of crosslinking [55]:

(6) $$\nu =-\frac{\ln\left(1-\vartheta_{2m}\right)+\vartheta_{2m}+\chi_{12}\vartheta_{2m}^{2}}{V_{1}\left(\vartheta_{2m}^{\frac{1}{3}}-\frac{\vartheta_{2m}}{2}\right)},$$

where ν is a degree of crosslinking of the elastomer; $V_{1}$ is the molar volume of the solvent (106.5 cm^3^/mol for toluene); $\vartheta_{2m}$ is the molar fraction of the polymer for the equilibrium swelling; $\chi_{12}$ is the Flory–Higgins coefficient, which characterizes the polymer-solvent interaction and is equal to 0.393 for the analyzed system [56,57]. The calculation results are presented in Table 2.

From the data obtained, it follows that a sample of composite elastomer cured in the absence of an external electric field has a lower content of unbound components, a lower degree of swelling, and a higher degree of crosslinking of polymer molecules. In turn, vulcanization of the composite polymer in an electric field results in a material with a relatively low degree of crosslinking and contains organic components that are not connected to a common polymer network. These features are consistent with data characterizing the electrorheological behavior of composite elastomers and their dielectric properties.

**Figure 8:**
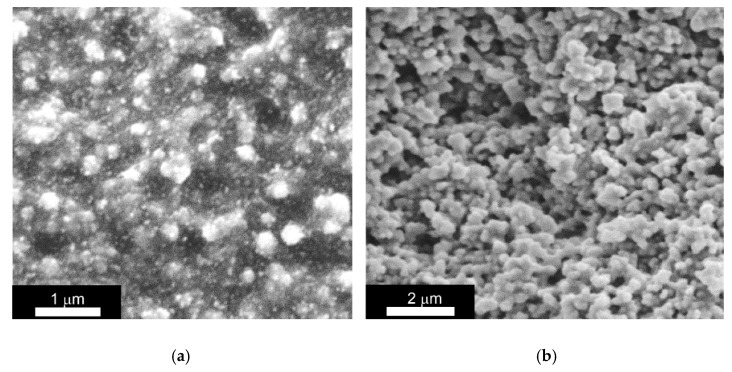
Scanning electron microscopy data for composite elastomers (**a**) ERE-0 and (**b**) ERE-5 after analysis of the degree of crosslinking of the polymer matrix (after washing in toluene and ethanol).

The analysis of ERE-0 and ERE-5 samples after determining the degree of swelling (washed with toluene and ethanol), by scanning electron microscopy (Figure 8), showed that the microstructure of the former sample was characterized by the presence of TiO_2_ particles immobilized in a continuous polymer matrix. Conversely, the surface of the ERE-5 sample after washing acquired a loose structure, indicating a partial destruction of the polymer matrix.

Based on the combined results obtained, one can conclude that the ERE-0 sample was an elastomeric composite with filler particles uniformly distributed in a polymer matrix, characterized by a high degree of crosslinking. In addition to the crosslinked polymer, the polymer matrix of the ERE-5 composite included about 10% of the organic component that was not bound with the polymer matrix.

Apparently, during the curing of the elastomer in an external electric field, a partial segregation of the hardener, which has a higher dielectric constant compared to oligomers of polydimethylsiloxane, occurred. This resulted in its localization near the polarized particles of titanium dioxide, so the curing of the elastomer occurred non-uniformly and a fraction of the organic molecules was not bound into a polymer matrix. This effect can be used to obtain elastomeric composites with a controlled elasticity and creates the background for the design of highly effective electrorheological elastomers.

## 4. Conclusions

The viscoelastic and dielectric properties of silicone elastomers filled with isotropic particles of hydrated titanium dioxide with 100–200 nm size and cured in, and in the absence of, an electric field were compared. An analysis of the dependences of the storage modulus and the loss modulus of filled elastomers made it possible to reveal the effect of the electric field on the Payne effect in electrorheological elastomers. An elastomer vulcanized in an electric field showed high values of electrorheological sensitivity, which reached 250% for the storage modulus and 1100% for the loss modulus.

It was found that it is necessary to bring the elastomer to a pre-stressed state by applying an external tangential load in order to achieve high electrorheological sensitivity of the composite elastomeric system.

It has been shown, for the first time, that the vulcanization of filled elastomers in an electric field can lead to a significant decrease in the degree of crosslinking in the elastomer. This effect should be taken into account in the design of electroactive elastomeric materials.

## Figures and Tables

**Table 1:** The results of fitting the dielectric spectra of composite elastomer samples to Havriliak–Negami equation.

Sample Name	$\varepsilon_{S}$	$\varepsilon_{\infty }$	$\varepsilon_{\infty }-\varepsilon_{S}$	*τ*	*α*	*β*
ERE-0	8.9	4.0	4.9	2.4⋅10^–5^	0.85	0.80
ERE-5	37.7	5.7	32.0	5.6⋅10^–4^	0.85	0.80

**Table 2:** The results of swelling and crosslinking degree calculations for the samples of composite elastomers based on polydimethylsiloxane and titanium dioxide, cured in, and in the absence of, an electric field.

Sample Name	*β*, %	*ω*_c_, %	*ν*, mol/g
ERE-0	170	3.5	0.0056
ERE-5	296	10.3	0.0013

## Supplementary Materials

The following are available online at https://www.mdpi.com/2073-4360/12/9/2137/s1.
