# Supplementary material for: Electrorheological Properties of Polydimethylsiloxane/TiO2-Based Composite Elastomers
Source: Polymers (Basel). 2020 Sep 18;12(9):2137. doi: 10.3390/polym12092137 (PMC7569977; doi:10.3390/polym12092137)
Supplement: Supplementary file 1 [file polymers-12-02137-s001.pdf]

Supplementary Information

Electrorheological Properties of Polydimethylsiloxane/TiO_2-Based Composite Elastomers

Alexander V. Agafonov [1,*], Anton S. Kraev [1], Alexander E. Baranchikov [2] and Vladimir K. Ivanov [2,3]

1 Krestov Institute of Solution Chemistry, Russian Academy of Sciences, Ivanovo, 153045, Russia; a.s.kraev@mail.ru

2 Kurnakov Institute of General and Inorganic Chemistry, Russian Academy of Sciences, Moscow, 119991, Russia; a.baranchikov@yandex.ru (A.E.B.); van@igic.ras.ru (V.K.I.)

3 Higher School of Economics, National Research University, Moscow, 101000, Russia

* Correspondence: ava@isc-ras.ru

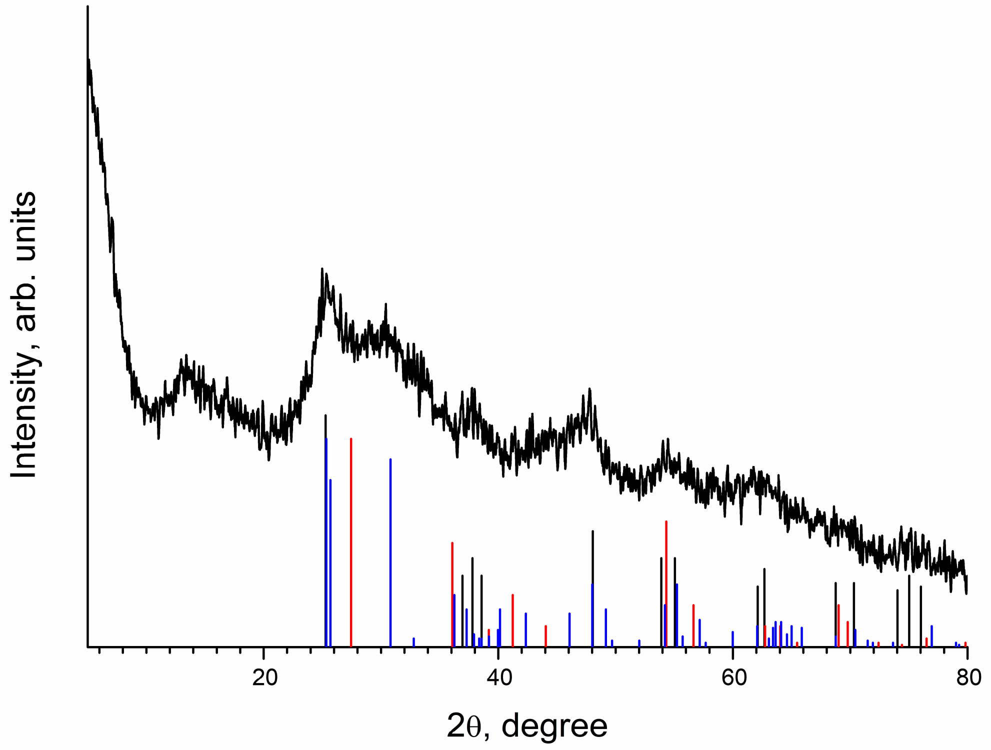

Figure S1. X-ray diffraction pattern of titanium dioxide powder obtained by hydrolysis of titanium isopropoxide in ethanol. Bragg peak positions for anatase are given in black, Bragg peak positions for rutile are given in red, Bragg peak positions for brookite are given in blue.

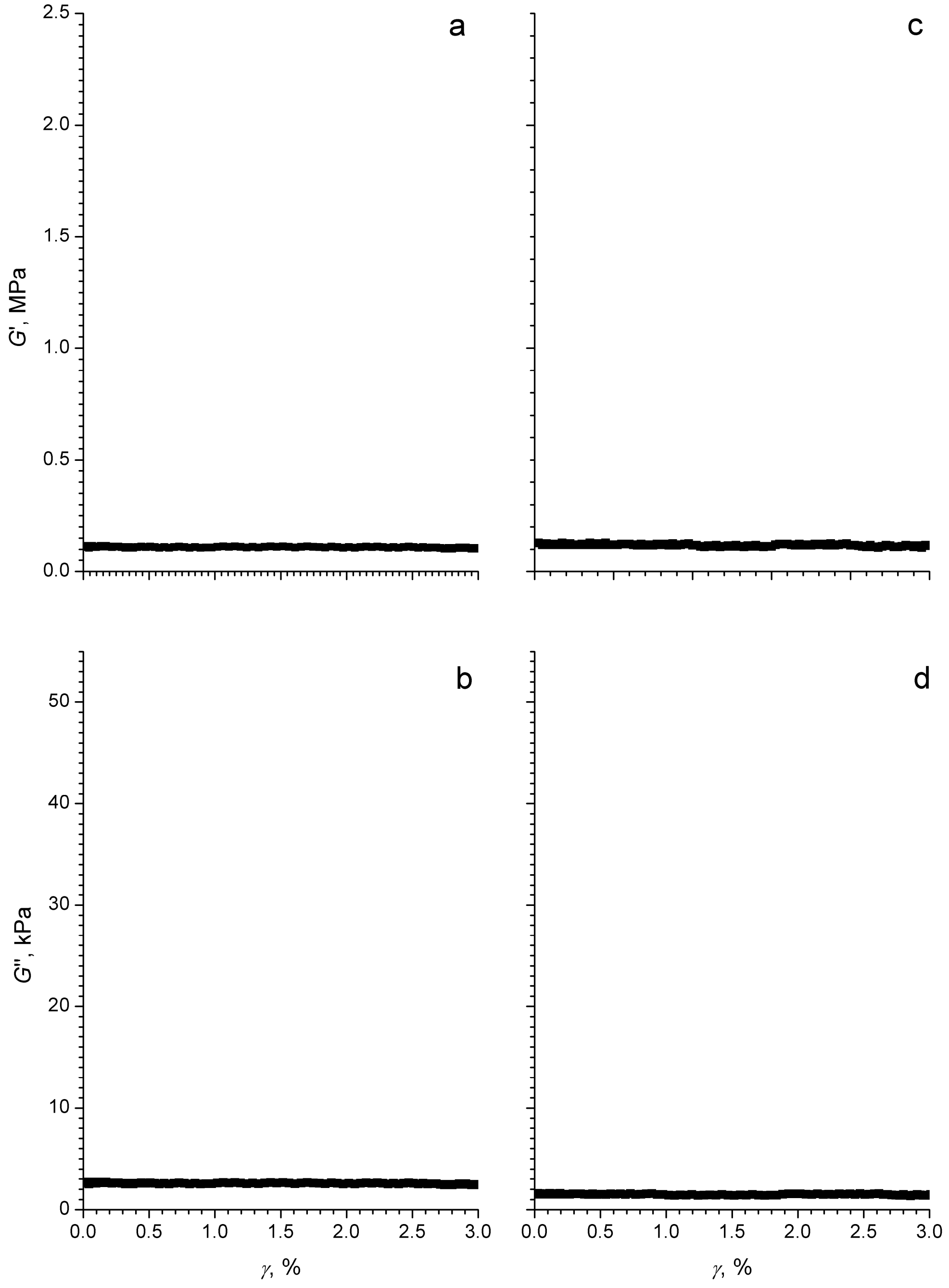

Figure S2. (**a**, **c**) Storage modulus (G') and (**b**, **d**) loss modulus (G'') as functions of relative deformation for non-filled elastomer samples vulcanised (**a**, **b**) in the absence of an electric field or (**c**, **d**) in an electric field. The shear rate was 0.1 rad/s.

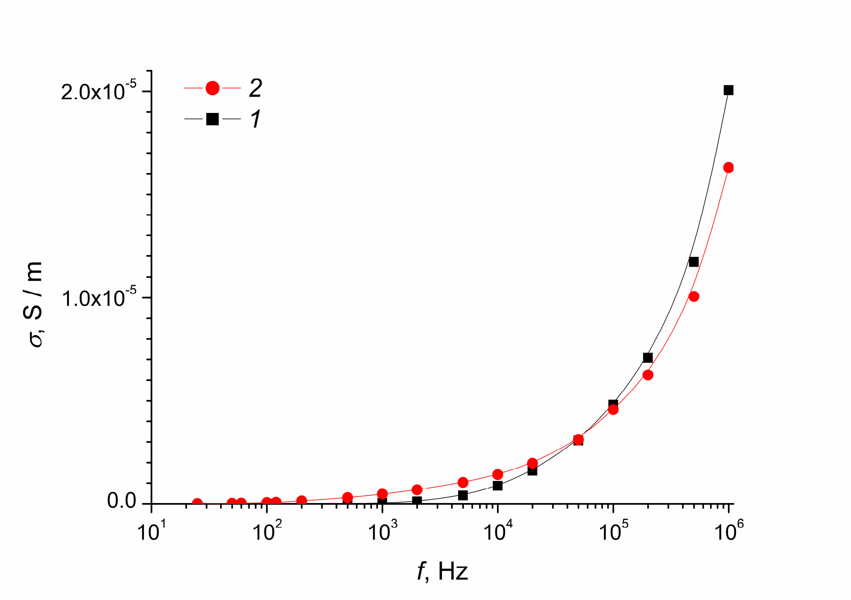

Figure S3. Conductivity of the (*1*) ERE-0 and (*2*) ERE-5 samples vs frequency.
